# COVID-19 and Its Aftermath on Pediatric Oral Health: A Study of Dental Caries and Hygiene in Romanian Children

**DOI:** 10.3390/children12081061

**Published:** 2025-08-12

**Authors:** Maximilian Ilea, Alina-Ioana Forray, Nausica Bianca Petrescu, Ioana-Codruta Mirica, Alina Ormenişan, Mine Betül Üçtaşli, Adriana Melnic, Ondine Patricia Lucaciu

**Affiliations:** 1 Department of Oral and Maxillofacial Surgery, Faculty of Dental Medicine, “George Emil Palade” University of Medicine, Pharmacy, Science & Technology of Târgu Mureş, 540139 Târgu Mureș, Romania; ilea.maximilian.21@stud.umfst.ro (M.I.); alina.ormenisan@umfst.ro (A.O.); 2 Department of Community Medicine, “Iuliu Hațieganu” University of Medicine and Pharmacy, 400012 Cluj-Napoca, Romania; alina.forray@umfcluj.ro; 3 Department of Oral Health, “Iuliu Hațieganu” University of Medicine and Pharmacy, 400012 Cluj-Napoca, Romania; mirica.ioana@umfcluj.ro (I.-C.M.);; 4 Department of Restorative Dentistry, Faculty of Dentistry, Gazi University, Ankara 06490, Turkey; uctasli@gazi.edu.tr; 5 National Institute of Public Health in Romania, 050463 Bucharest, Romania; adriana.melnic@insp.gov.ro; 6 Romanian Cancer Society, 400090 Cluj-Napoca, Romania

**Keywords:** dental caries, oral health, pediatric dentistry, COVID-19, health behavior, cariogenic diet, DMFT index, cross-sectional study, Romania

## Abstract

**Highlights:**

**What are the main findings?**

- A four-fold rise in affected teeth in 6-year-old rural children’s permanent teeth post-pandemic (29.6% vs. 6.8%) was noted.
- Despite increased toothbrushing (70% vs. 26% twice daily), a more cariogenic diet and shift from preventive to pain-driven visits offset this.

**What is the implication of the main finding?**

- Negative oral health trends highlight the need for Romania to implement strong public health programs focusing on nutrition education and proactive dental care.
- For pediatric health policy, promoting personal hygiene alone is insufficient; it must be coupled with strategies addressing dietary habits and access to professional dental care.

**Abstract:**

**Background/Objectives**: Official data on the oral health of young children in Romania are limited, especially concerning the societal shifts following the COVID-19 pandemic. This study aimed to compare the oral health status, hygiene habits, diet, and dental care patterns of 6-year-old children in a rural Romanian region before and after the pandemic. **Methods**: A cross-sectional survey studied two groups of 6-year-olds from rural Transylvania: Group 1 (*n* = 77), assessed 2018–2020 pre-pandemic, and Group 2 (*n* = 136), assessed in 2024 post-pandemic. Clinical data used the Decayed, Missing, and Filled Teeth (DMFT) index. Parents completed questionnaires on oral hygiene, diet, dental visits, and pandemic-related topics for Group 2. Chi-square, Student’s *t*-tests, and a multiple linear regression were used to analyze the data. **Results**: Post-pandemic, 70% of children brushed twice daily, up from 26%. Despite this, negative outcomes increased: the number of affected teeth rose from 6.8% to 29.6% (*p* < 0.001), sugar intake increased, and dental visits became more reactive, with pain being the main reason for 61% of post-pandemic visits, compared to 17% pre-pandemic. **Conclusions**: This study reveals a significant oral health paradox: despite a three-fold increase in recommended toothbrushing, caries experience in permanent teeth increased four-fold. This outcome was driven by a high-risk environment of more cariogenic diets and reduced preventive care, with lower maternal education also identified as a key independent risk factor. These findings show that promoting hygiene alone is insufficient, underscoring the urgent need for public health programs that integrate nutritional counseling, improve access to preventive care, and address underlying socioeconomic disparities.

## 1. Introduction

### 1.1. The Global Scale and Economic Burden of Early Childhood Caries

Oral health is an integral and inseparable component of overall health, yet early childhood caries (ECC) persists as a significant and inadequately addressed global public health crisis [1]. Fundamentally, dental caries is an infectious, bacteria-mediated disease, primarily caused by the interaction of oral bacteria such as *Streptococcus mutans* and *Lactobacillus* spp. with fermentable carbohydrates from the diet. Beyond the immediate oral effects of pain and infection, the consequences of untreated caries can have systemic implications. A growing body of evidence links the chronic inflammation and bacterial load from poor oral health to the exacerbation of various systemic diseases, including cardiovascular disease, diabetes mellitus, respiratory infections, and even certain cancers [2,3]. The World Health Organization has identified dental caries as the most common non-communicable disease worldwide, and its impact on pediatric populations is particularly severe [1]. According to a landmark report from the U.S. Surgeon General, dental caries is the single most common chronic disease of childhood, occurring at a rate five times more frequently than asthma among children and adolescents [4]. This high prevalence is not confined to a single nation but is a widespread international phenomenon. A systematic review and meta-analysis of global data on ECC confirms that in many regions, the prevalence in preschool-aged children frequently exceeds 70%. However, significant variation exists based on geography and socioeconomic conditions [5]. Indeed, high prevalence rates are consistently reported in cross-sectional studies globally, particularly in many low- and middle-income countries [6,7,8,9].

The consequences of such a high prevalence extend beyond individual health, placing a substantial and growing burden on national healthcare systems [1]. Global spending on oral healthcare, including both public and private sources, has reached approximately USD 387 billion, although the allocation is highly uneven across different regions and nations [10]. High out-of-pocket costs and catastrophic expenses prevent many from seeking oral healthcare. In 2015, dental issues cost approximately USD 357 billion in treatment and USD 188 billion in productivity losses worldwide, with disparities among countries of different income levels [11].

The management of ECC often requires high-tech, interventionist care, which consumes significant financial resources, especially in high-income countries. This economic strain is compounded by shortages of appropriately trained oral healthcare personnel and systemic barriers that hinder the implementation of effective, widespread prevention programs [1,12]. As a result, healthcare systems are often locked in a cycle of reactive, costly treatment rather than cost-effective prevention, struggling to manage the sheer volume of need presented by the nearly 530 million children globally who suffer from untreated dental caries in their primary teeth [1].

### 1.2. The Role of Socioeconomic Gradients and Social Determinants

The immense burden of ECC is not distributed equally across populations. Its prevalence is strongly and inversely correlated with national income levels and a range of interconnected socioeconomic factors [5]. A clear socioeconomic gradient is well-documented in the literature, demonstrating that children from higher-income countries and more affluent families have significantly better oral health outcomes. A comprehensive systematic review found that children in high-income countries may have up to 90% lower odds of experiencing poor oral health compared to their counterparts in low- or middle-income nations [13]. This disparity is starkly evident within the European continent, where a clear demarcation exists between wealthier Western European nations, which report lower caries rates, and less wealthy Eastern European nations, like Romania, where the prevalence of dental caries remains particularly high [13,14].

These inequities are driven by a complex interplay of social determinants of health, which operate at the individual, family, and community levels [15]. Among the most influential of these is parental education. A large body of evidence consistently shows that lower parental education levels are a significant risk factor for higher caries prevalence and poorer oral health behaviors in children [16,17]. The educational attainment of the mother, in particular, has been identified as a powerful predictor of a child’s oral health status, influencing factors such as dietary habits, oral hygiene practices, and the utilization of preventive and restorative dental care [17]. For families residing in rural areas, the specific focus of the present study, these challenges are often magnified. Rural populations frequently face a unique set of persistent barriers to accessing pediatric dental care, including prohibitive out-of-pocket costs, lack of adequate insurance coverage, long travel distances to dental facilities, and a scarcity of pediatric dental providers [14,18].

### 1.3. The COVID-19 Pandemic: A Dual Threat to Pediatric Oral Health

The COVID-19 pandemic, beginning in early 2020, introduced an unprecedented disruption to this already challenging landscape, creating a dual threat to pediatric oral health. The first threat was the profound interruption of routine dental care. Globally, the pandemic triggered a significant and abrupt decrease in the utilization of preventive dental services for young children [19,20]. In the United States, for example, the proportion of children who had a dental visit in the past year fell from 82.6% to 78.2%, with preventive services experiencing the sharpest decline [20]. Similar trends were reported internationally; a study in Saudi Arabia documented a drop of nearly 40% in pediatric dental patient flow during the pandemic period [21]. This reduction in care was not only due to systemic lockdowns and clinic closures but was also fueled by widespread parental hesitancy, as caregivers delayed or avoided appointments due to fears of infection and other logistical challenges [22]. While innovative solutions like teledentistry emerged to provide triage and ensure some continuity of care [23,24], its effectiveness was limited by challenges related to digital infrastructure and access, particularly in low-resource and rural communities. Teledentistry ultimately served as a valuable supplement but could not replace the necessity of in-person clinical examinations and preventive procedures [24,25].

The second simultaneous threat was a significant and detrimental shift in children’s daily health behaviors. Numerous studies have documented that lockdown measures led to negative changes in dietary habits, creating a more cariogenic environment within the home [26,27]. A clear and consistent trend emerged of increased consumption of high-calorie snacks, sweets, junk food, and sugary drinks [26,27]. This shift was attributed to a confluence of factors, including increased access to unhealthy foods at home, boredom from confinement, emotional eating as a coping mechanism for stress, and a general relaxation of parental rules regarding diet during a period of crisis [27,28]. This combination of reduced professional preventive care and increased daily exposure to cariogenic diets created a high-risk “perfect storm” environment, primed for the initiation and progression of dental caries in young, vulnerable children.

This situation is particularly acute in Romania, where a national public oral health program for children is not robustly implemented, and access to preventive care, especially in rural areas, is largely dependent on private practitioners and out-of-pocket payments [14]. In Romania, the beginning of 2020 was marked by the outbreak of the SARS-CoV-2 pandemic, which altered the population’s perception and behavior regarding dental treatments and quality of life [29]. During the state of emergency, the dental offices were officially closed, with only a few remaining open for emergency care [30]. Although the pandemic has ended, it has changed perceptions regarding dental treatments. Specifically, anxiety has risen. The financial status of the population was affected, and the belief that oral health can be postponed has changed [29].

### 1.4. Rationale and Aims of the Current Study

The existing literature clearly establishes the high global burden of ECC, its deep roots in socioeconomic disparities, and the dual pandemic-driven threats of reduced preventive care and increased sugar consumption. We know from robust evidence that early and regular preventive dental visits are highly effective, significantly reducing future caries incidence and promoting long-term oral health [31,32]. The widespread disruption to these visits is therefore a major public health concern. However, a critical gap remains in our understanding of the combined, quantifiable clinical impact of these pandemic-era trends within specific, high-risk populations.

Particularly in a setting like rural Romania, a region characterized by a high pre-existing caries burden, documented socioeconomic challenges, and known barriers to accessing care [14,33,34], the net clinical consequences of the pandemic have not been directly measured. A key unanswered question, therefore, is how competing factors, such as potential improvements in home hygiene and documented risks like increased sugar intake and reduced professional care, have collectively influenced clinical outcomes in a high-risk pediatric population. Therefore, this study was designed to fill this critical knowledge gap by providing a direct comparison of the oral health status of 6-year-old children in this vulnerable region, both before and after the pandemic. The primary objective was to assess the relationship between the COVID-19 period and observed changes in dental caries prevalence, oral hygiene habits, nutrition, and dental care utilization patterns. Based on the documented dual threats of increased sugar consumption and reduced professional care during the pandemic, we hypothesized that despite any potential improvements in home oral hygiene, the net effect would be a significant increase in the prevalence of dental caries in the permanent dentition of the post-pandemic cohort.

## 2. Materials and Methods

### 2.1. Study Design and Ethical Considerations

A comparative, cross-sectional oral health survey was conducted in rural pediatric populations from Cluj County, in the Transylvania region of Romania. The study aimed to compare the oral health status between two distinct cohorts of 6-year-old children assessed at different time points: one pre-pandemic and one post-pandemic. The study protocol was approved by the relevant local (school), regional (school inspectorate) (3406/1/15 April 2024), and national authorities (Ministry of Health from Romania, Approval No. 3411/5 April 2018), as referenced in the initial cohort’s publication [33] and Ethical Committee of George Emil Palade University of Medicine, Pharmacy, Science and Technology of Târgu Mures 3851/14 July 2025. Prior to any examination, written informed consent was obtained from the parents or legal guardians of all participating children, in compliance with the Declaration of Helsinki.

### 2.2. Study Population and Sampling Strategy

The study’s comparative cross-sectional design necessitated the of two different sampling strategies, a key methodological consideration. The goal was to recruit two cohorts of 6-year-old children from rural schools in Cluj County that were as comparable as possible, despite the logistical challenges of the pre- and post-pandemic periods.

Group 1 (pre-pandemic cohort) consisted of 77 children from the Cluj County examined between 2018 and 2020 as part of a national oral health project. The sampling for this cohort followed the World Health Organization (WHO) Pathfinder survey methodology, a stratified cluster sampling approach designed to obtain representative data. The detailed application of this methodology has been described in a previous publication [33].

Group 2 (post-pandemic cohort) consisted of 136 children examined in 2024. Replicating the WHO Pathfinder methodology was not feasible due to logistical and resource constraints in the post-pandemic environment. Therefore, a pragmatic multi-stage convenience sampling method was employed. Initially, a list of accessible rural schools within Cluj County was compiled. Subsequently, school headmasters were contacted to present the study’s objectives and procedures. All schools that expressed a willingness to participate were included in the sample. Within these consenting schools, parents of all 6-year-old children were invited to informational meetings where the study was explained. All children whose parents or legal guardians provided written informed consent were enrolled in the study. While the sampling methods differed, efforts were made to enhance cohort comparability and internal validity by matching the groups on key criteria: age (6 years), gender balance, location (rural schools), and geographical region (Cluj County).

### 2.3. Examiner Calibration

To ensure diagnostic consistency, all examiners involved in the study underwent a calibration process based on WHO guidelines (WHO, 2013). For Group 1, the calibration of examiners for the International Caries Detection and Assessment System (ICDAS) was conducted as detailed by Lucaciu et al. (2020) [33], achieving a Cohen’s kappa value of 0.75.

For Group 2, four experienced dentists were calibrated for the direct application of the DMFT index. In accordance with WHO (2013) criteria [35], a tooth was recorded as decayed (D) only when a lesion presented with an “unmistakable cavity, undermined enamel, or a detectably softened floor or wall” upon gentle probing. Inter-examiner reliability for the examining team was established once a Cohen’s kappa value of 0.85 was achieved. The final clinical examinations for Group 2 were performed by two of the four calibrated dentists.

### 2.4. Data Collection and Diagnostic Criteria

Clinical examinations for both groups were conducted on school premises in a seated position under natural light, aided by a plane dental mirror and a ball-tipped WHO Community Periodontal Index (CPI) probe for gentle tactile assessment.

For Group 1, clinical data on caries status were collected using the two-digit ICDAS II coding system, which records both the restorative status and the severity of the carious lesion. For Group 2, caries status was assessed directly using the Decayed, Missing, and Filled Teeth (DMFT) index.

In addition to the clinical examination, parents of all participants completed a structured questionnaire. The baseline instrument, based on WHO recommendations, collected data on parental education level, the frequency of and reasons for the child’s dental visits, oral hygiene habits (e.g., frequency of cleaning, tools used), and dietary patterns, with a focus on the consumption frequency of nine categories of sugary foods and drinks. To minimize misunderstanding and improve data quality, the questionnaires were completed in the presence of a research team member who could provide clarification on the questions as needed. The questionnaire administered to both groups, based on WHO recommendations for national surveys, was divided into the following sections: (1) Level of education for the father and mother (or other legal guardians); (2) Frequency of the child’s toothache or discomfort, number of dental visits in the last year, and the reason for the most recent dental visit (e.g., pain, check-up, treatment); (3) Frequency of teeth cleaning, types of tools used (e.g., toothbrush, toothpicks, dental floss), and whether toothpaste is used; and (4) A food frequency questionnaire assessing the consumption of nine specific categories of foods and drinks: fresh fruit; biscuits, cakes, cream, sweet pies, buns; sweetened soft drinks; sweetened fruit juices; honey; chewing gum containing sugar; sweets/candy; milk with sugar/honey; and cocoa with sugar/honey. Parents rated the frequency on a five-point scale from “never” to “a few times a day.”

For the Group 2 cohort, this questionnaire was supplemented with seven additional questions designed to assess the perceived impact of the COVID-19 pandemic (2020–2022). This following section queried parents on the following: (1) Difficulties in accessing dental services when needed since 2020; (2) Financial challenges since the pandemic affecting their ability to prioritize the child’s dental care; (3) Changes in the child’s consumption of sugary snacks and drinks compared to the pre-pandemic period; (4) The development or worsening of stress-related oral habits (e.g., teeth grinding, nail biting) and whether these habits persist; (5) Changes in the frequency of the child’s dental visits compared to the pre-pandemic period; (6) Changes in the child’s level of fear of going to the dentist; and (7) Whether they believed dental problems in the past year were influenced by pandemic-related delays in care.

### 2.5. Data Transformation for Comparative Analysis

To facilitate a direct comparison between the two groups, the detailed ICDAS II data from Group 1 were systematically converted into DMFT scores. The full conversion protocol, which maps each ICDAS code to its corresponding DMFT classification with a detailed rationale, is presented in Table 1. Our approach defines a tooth as “Decayed” (D) at the threshold of ICDAS code ≥3. This is a validated method that balances diagnostic sensitivity with the need for comparability with traditional epidemiological surveys that use a cavitation-based index [36,37]. While this conversion sacrifices some sensitivity for non-cavitated lesions (ICDAS 1–2), it ensures that the resulting DMFT data are robust and comparable.

### 2.6. Statistical Analysis

Data were entered into a database and all statistical analyses were performed using IBM SPSS Statistics for Windows (Version 28.0, IBM Corp., Armonk, NY, USA). Descriptive statistics, including means, standard deviations (SD), frequencies, and percentages, were calculated to summarize the sample characteristics. For inferential analysis, several tests were employed depending on the nature of the data and the research question.

The chi-square (χ^2^) test was used to compare categorical variables between the two cohorts, such as the proportions of decayed, missing, and filled teeth. Independent samples *t*-tests were used to compare the means of continuous variables between the two groups, such as the overall dmft/DMFT scores and sweets consumption. To assess the monotonic relationship between ordinal variables, Spearman’s rank-order correlation (ρ) was used. The Kruskal–Wallis H test, a non-parametric analysis of variance, was used to compare caries scores across the multiple levels of parental education. Finally, a multiple linear regression was performed to simultaneously assess the predictive value of the study cohort, maternal education, sweets consumption, and brushing frequency on the permanent dentition DMFT score. For all statistical tests, a *p*-value of less than 0.05 was considered significant.

## 3. Results

### 3.1. Sample Characteristics

The final study population consisted of 213 children from two distinct cohorts: the pre-pandemic Group 1 (2018–2020), with a sample size of 77 children (*n* = 77), and the post-pandemic Group 2 (2024), with a sample size of 136 children (*n* = 136). The demographic characteristics of each cohort were analyzed to provide context for the clinical findings. The gender distribution showed slight differences between the groups. In Group 1, females constituted a majority of the sample at 57% (*n* = 44), while males accounted for 43% (*n* = 33). Conversely, Group 2 had a slight male majority, with males constituting 52% (*n* = 71) and females 48% (*n* = 65) of the cohort.

### 3.2. Clinical Caries Assessment: DMFT Comparison Between Groups

A comparison of caries experience revealed significant and contrasting patterns of disease between the two cohorts in both the primary and permanent dentitions. A detailed breakdown of the mean caries experience per child is presented in Table 2, while a tooth-level analysis of caries prevalence and index composition is shown in Table 3. In the primary dentition, children in Group 1 (2020 cohort) demonstrated a significantly higher overall prevalence of affected teeth. An analysis of all primary teeth examined showed that 43.3% were affected by decay, extraction, or fillings, a figure substantially higher than the 26.8% observed in Group 2 (2024 cohort), with this difference being statistically significant (*p* < 0.001). This elevated disease burden in the earlier cohort was primarily driven by untreated decay. The Decayed (D) component constituted 97.0% of all affected primary teeth in Group 1, a rate significantly higher than the 87.2% observed in Group 2 (χ^2^ = 32.91, *p* < 0.001). Conversely, indicators of dental treatment and disease sequelae showed an inverse trend. The proportion of missing (M) primary teeth due to caries was significantly higher in Group 2, representing 7.3% of affected teeth compared to just 2.7% in Group 1 (χ^2^ = 10.99, *p* = 0.001). Similarly, restorative care, represented by the Filled (F) component, was notably more common in the 2024 cohort. In Group 2, fillings accounted for 5.4% of affected primary teeth, a twenty-fold increase compared to the mere 0.2% observed in Group 1 (χ^2^ = 11.69, *p* < 0.001).

**Figure 1:**
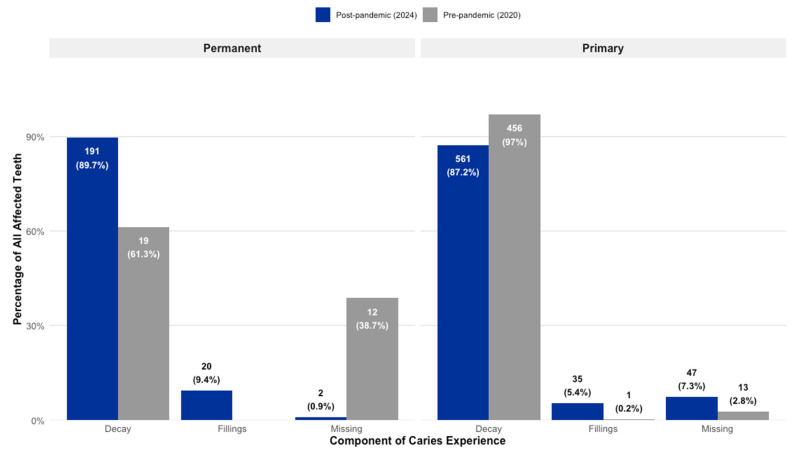
Comparison of tooth-level caries prevalence and the composition of the dmft/DMFT index in primary and permanent dentition, by study cohort.

In the permanent dentition, a striking reversal of this pattern was observed. The 2024 cohort (Group 2) exhibited a significantly higher proportion of affected permanent teeth, at 29.6%, compared to only 6.8% in the 2020 cohort (Group 1), a highly significant difference (*p* < 0.001), as shown in Table 3. This increased disease prevalence in the later cohort was largely attributable to the decay (D) and filled (F) components. In Group 2, 89.7% of affected permanent teeth were decayed, and a notable 9.4% were filled. This stands in stark contrast to Group 1, which had a lower proportion of decayed permanent teeth (61.3%) and, critically, no recorded fillings in the permanent dentition. The most dramatic difference was observed in the missing (M) component; in Group 1, tooth loss accounted for a substantial 38.7% of all affected permanent teeth. In Group 2, the count of missing permanent teeth was only two (*n* = 2), a number too low to permit meaningful statistical comparison. A graphical overview providing a general summary of these clinical findings is presented in Figure 1.

### 3.3. Association of Oral Health Status with Questionnaire Data

#### 3.3.1. Parental Education Level

**Figure 2:**
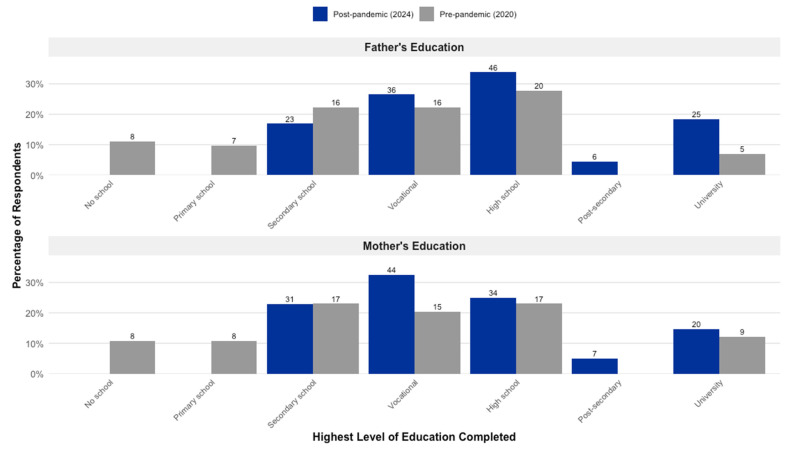
Distribution of parental education levels for the pre-pandemic and post-pandemic cohorts.

The educational attainment of parents, a key socioeconomic indicator, differed considerably between the two cohorts (Figure 2). In Group 2, a higher proportion of parents reported completing high school or university studies compared to their counterparts in Group 1. Specifically, 34% of fathers and 25% of mothers in the 2024 cohort had completed high school, compared to 26% of fathers and 21% of mothers in the 2020 cohort. This trend was also evident at the university level, with 18% of fathers and 15% of mothers in Group 2 holding a university degree, compared to 8% and 12%, respectively, in Group 1.

To formally test the association between parental education and clinical outcomes, a series of Kruskal–Wallis tests were performed. The results indicated a statistically significant association between father’s education level and both the primary tooth caries score (dmft, H(6) = 13.35, *p* = 0.038) and the permanent tooth caries score (DMFT, H(6) = 18.04, *p* = 0.006). Mother’s education level was also significantly associated with the permanent tooth score (DMFT, H(6) = 16.10, *p* = 0.013), but not the primary tooth score (dmft, *p* = 0.144). Pairwise comparisons for father’s education revealed that children of university-educated fathers had significantly lower DMFT scores than those whose fathers had a high school education (*p* = 0.002).

Furthermore, to directly test the relationship between parental education and oral health behaviors, Spearman’s rank-order correlations were performed. The analysis confirmed a statistically significant, albeit small, positive correlation between brushing frequency and both maternal education (ρ = 0.154, *p* = 0.026) and paternal education (ρ = 0.198, *p* = 0.004).

#### 3.3.2. Consumption of Sweets

Analysis of dietary patterns, based on parental responses to the questionnaire, revealed a significant difference in the consumption of sugary foods and drinks between the two cohorts. As detailed in Table 4, the 2024 cohort reported a statistically higher frequency of consumption for nearly all cariogenic items compared to the 2020 cohort. The most pronounced differences were seen in the consumption of “fresh fruit,” “cookies, cakes…,” and “sweets/candies,” all of which were significantly higher in the 2024 cohort (*p* < 0.001). The overall mean sweets consumption score per child was also significantly higher in the 2024 cohort (2.36 ± 0.031) compared to the 2020 cohort (1.99 ± 0.079). However, a Spearman’s correlation analysis found no statistically significant relationship between the individual mean sweets intake score and the caries scores for either the permanent teeth (DMFT score, ρ = 0.023, *p* = 0.748) or the primary teeth (dmft score, ρ = −0.025, *p* = 0.726).

#### 3.3.3. Oral Hygiene and Dental Service Utilization

##### Effect of Oral Hygiene on Oral Health

**Figure 3:**
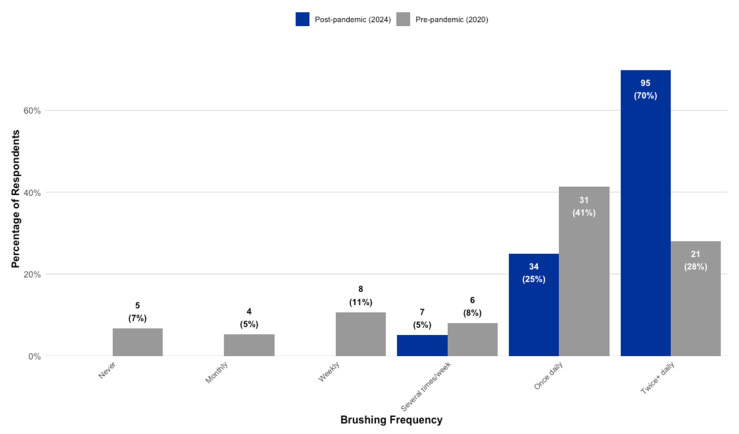
Comparison of self-reported toothbrushing frequency between the pre-pandemic and post-pandemic cohorts.

A comparison of parental reports revealed significant behavioral shifts between the two cohorts. First, there was a three-fold increase in optimal oral hygiene practices, with the proportion of children brushing at least twice daily rising from 28% (*n* = 21) in the pre-pandemic cohort to 70% (*n* = 95) in the post-pandemic cohort (Figure 3).

Second, this improvement in hygiene occurred alongside a significant and detrimental shift in diet. Independent samples *t*-tests confirmed that the post-pandemic cohort consumed significantly more sugary items than their pre-pandemic counterparts. This was true for children brushing once daily (t(60.92) = −4.16, *p* < 0.001, Cohen’s d = 1.04) and for those brushing twice or more daily (t(39.34) = −4.44, *p* < 0.001, Cohen’s d = 0.87), with both differences representing large effect sizes. The clinical data, presented in Table 5, were analyzed to understand how these competing behavioral changes impacted caries experience. In the pre-pandemic cohort, more frequent brushing showed a positive trend. Children who brushed at least twice daily had lower mean dmft scores (M = 4.67, SD = 4.56) than those who brushed once daily (M = 6.74, SD = 3.34); however, this difference did not reach statistical significance, t(34.12) = 1.79, *p* = 0.083. Notably, the effect size for this trend was medium (Cohen’s d = 0.54). In the post-pandemic cohort, this protective trend vanished. There was no significant difference in caries experience between brushing groups in either the primary dentition (t(62.25) = −0.68, *p* = 0.501) or the newly erupted permanent dentition (t(66.33) = −0.62, *p* = 0.536).

To simultaneously assess the predictors of caries experience in the permanent dentition, a multiple linear regression was performed. The analysis entered four predictor variables into the model: study cohort, maternal education level, sweets consumption score, and brushing frequency. The overall model was statistically significant, F(4, 173) = 16.77, *p* < 0.001, and explained 26.3% of the variance in DMFT scores (adjusted R^2^ = 0.263). The detailed results of the regression coefficients are presented in Table 6. The analysis identified two significant predictors of the DMFT score: being in the post-pandemic cohort was associated with a higher DMFT score, while a higher level of maternal education was associated with a lower DMFT score. In this model, brushing frequency and the sweets consumption score were not statistically significant predictors.

##### The Effect of Dental Services on Oral Health

**Figure 4:**
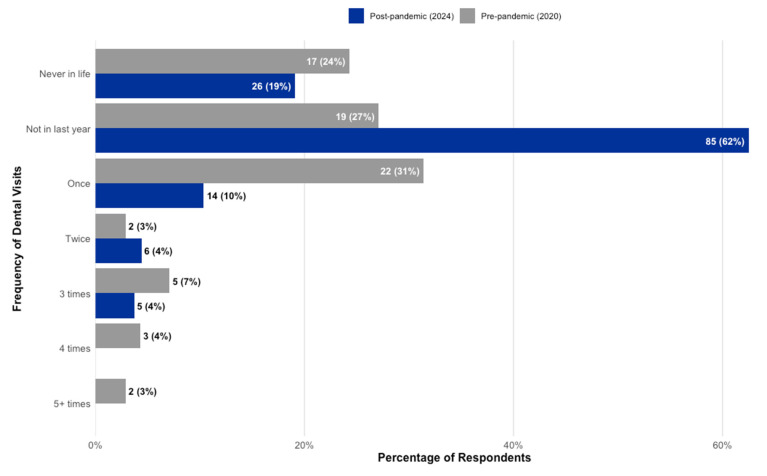
Comparison of self-reported frequency of dental visits in the past year between the pre-pandemic and post-pandemic cohorts.

Profound differences in the patterns of dental service utilization were observed between the two cohorts (Figure 4). The most striking finding was the high proportion of children in the 2024 cohort (Group 2) who had not accessed dental care in the preceding year. A significant majority of this group (63%, *n* = 86) reported not having been to the dentist in the last 12 months, a figure more than double the 25% (*n* = 19) reported for the 2020 cohort (Group 1). Conversely, children in the 2020 cohort reported more regular, if infrequent, visits. Nearly one-third of children in Group 1 (29%, *n* = 22) had visited the dentist once in the last year, compared to only 10% (*n* = 14) in Group 2. In total, 17% of children in Group 1 reported multiple visits (two or more) in the past year, compared to just 8% in Group 2.

**Figure 5:**
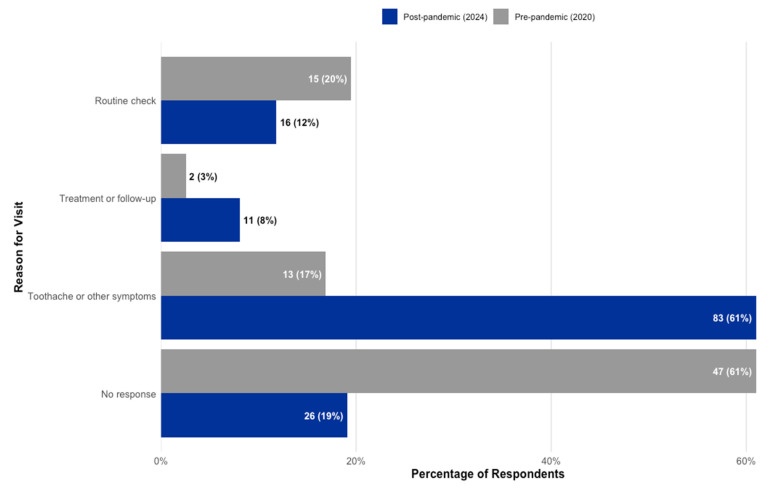
Comparison of self-reported reasons for the most recent dental visit, by study cohort.

The motivation for seeking dental care differed significantly between the two cohorts (χ^2^ = 38.70, *p* < 0.001), reflecting the shift in service utilization patterns. As shown in Figure 5, the primary driver for dental visits in the 2024 cohort (Group 2) was the experience of pain, which accounted for a majority of visits (61%, *n* = 83). In contrast, pain was a much less frequent reason for visits in the 2020 cohort (Group 1), accounting for only 17% (*n* = 13) of attendances. Conversely, routine check-ups were more common in the pre-pandemic period, making up 19% (*n* = 15) of visits for Group 1, compared to 12% (*n* = 16) for Group 2.

### 3.4. Perceived Impact of the COVID-19 Pandemic

Parental perceptions of the pandemic’s impact were assessed using a supplementary questionnaire administered to the post-pandemic cohort, with detailed results presented in Table 7. The findings reveal a significant disconnect between parental perception and the study’s clinical and dietary data. For instance, while a majority of parents (60.3%) believed their child’s sweets consumption had not changed, this perception contrasts sharply with the data indicating significantly higher sugar intake for this cohort (Table 2). Regarding access to care, most parents (73.4%) reported that financial challenges did not impede their child’s dental care; however, a substantial portion (34.6%) still experienced difficulties accessing dental services for other reasons. Importantly, among those parents who reported their child had recent dental problems, an overwhelming majority (87.2%) attributed these issues directly to pandemic-related delays.

## 4. Discussion

The principal finding of this study is the emergence of a paradoxical and concerning oral health landscape among 6-year-old children in rural Romania following the COVID-19 pandemic. While our post-pandemic cohort demonstrated significantly improved oral hygiene practices, this positive behavioral shift was critically undermined by a concurrent increase in cariogenic dietary habits and a substantial decline in preventive dental care. This resulted in a four-fold increase in caries experience in the newly erupted permanent dentition, a finding that is not only highly statistically significant (*p* < 0.001) but also profoundly clinically relevant, signaling a major public health challenge. This paper builds upon previous national data [33] by providing a focused comparison of pre- and post-pandemic periods at a critical stage in dental development, offering a granular view of the pandemic’s multifaceted impact.

Our findings of a significant post-pandemic increase in clinical caries are consistent with recent research from Greece, where a similar cohort of children showed a significant rise in dmft/DMFT scores following the lockdown period [38]. It is crucial to note that the primary drivers of this negative trend are not unique to Romania but reflect a near-universal phenomenon. The sharp decline in preventive dental care and the shift toward emergency, pain-driven visits documented in our cohort align perfectly with large-scale international findings. A major US study, for instance, found that pediatric dental care utilization dropped sharply during the pandemic, with a marked decrease in preventive services and a subsequent increase in more invasive procedures due to delayed treatment [39]. Our study builds on this by providing direct clinical evidence of the consequences of this service disruption—a key research gap, as much of the literature notes the disruption without being able to quantify the subsequent change in caries prevalence [40]. Therefore, while the trends observed in our study are consistent with a global pattern, the severity of the four-fold increase in caries may be particularly pronounced in our rural cohort, likely reflecting pre-existing systemic weaknesses in access to care, a situation common in middle-income countries where health systems struggled to maintain essential services during the pandemic [41]. Indeed, this interpretation is strongly supported by a recent systematic review of European countries that demonstrated a clear socioeconomic gradient, with higher national income being a powerful predictor of lower caries experience in children [13]. This provides a critical context for our findings, suggesting that the pandemic’s universal challenges had a disproportionately severe impact on a pediatric population already at high risk due to national economic conditions.

The central paradox observed, whereby improved toothbrushing frequency failed to confer a protective effect, is the most critical finding. This paradox is made even more striking when viewed in the context of international trends. While large-scale studies from both South Korea and Greece reported a general decline in oral hygiene practices during the pandemic [38,42], our younger, rural cohort reported a three-fold increase in recommended brushing. This contrast makes the subsequent four-fold rise in caries in our group particularly alarming. It is also worth noting the distinction between statistical significance and clinical relevance regarding the brushing habits in our pre-pandemic cohort. While the protective trend from more frequent brushing was not statistically significant (*p* = 0.083), its medium effect size (Cohen’s *d* = 0.54) suggests it was a clinically relevant phenomenon. This strongly suggests that in our cohort’s high-risk environment—characterized by a more cariogenic diet and reduced professional care—any potential benefits of improved brushing were overwhelmingly negated. This finding is crucial for public health messaging, as it demonstrates that promoting brushing alone is an insufficient strategy to prevent caries when broader dietary and systemic barriers remain unaddressed.

Sugar consumption is the primary cause of caries; reducing sugar, especially frequency of intake, is the most effective prevention strategy. Toothbrushing with fluoride is a key protective behavior that helps mitigate, but does not fully counteract, the effects of high sugar consumption. Ideally, both behaviors should be optimized, maintaining a low-sugar intake and brushing twice daily with fluoride toothpaste. In children, sugar restriction is slightly more impactful in caries prevention than brushing alone, but both are essential and synergistic [33,43]. The post-pandemic cohort reported a near three-fold increase in children brushing at least twice daily (70% vs. 26%). In isolation, this suggests successful public health messaging [44,45]. However, this cohort also reported significantly higher consumption of sugary foods and drinks. This finding aligns with global trends documented during the pandemic, where lockdowns and heightened stress were linked to negative dietary changes and emotional eating [46,47,48,49,50]. The scientific literature is clear that while brushing is crucial, its protective effects can be overwhelmed by high-frequency sugar exposure, which maintains a constantly acidic oral environment conducive to demineralization [51]. Our data compellingly suggest this is what occurred; the cariogenic challenge from the diet simply negated the benefits of more frequent mechanical plaque removal.

An important finding that adds nuance to our interpretation is the lack of a direct, statistically significant correlation between the composite mean score for sweets intake and the caries scores (dmft or DMFT) at the individual level (*p* > 0.7). This does not contradict the study’s main conclusion but rather refines it. It suggests that while the powerful group-level trends, including a significant increase in mean sugar consumption concurrent with a significant decrease in preventive care, provide a compelling ecological explanation for the rise in mean DMFT, the relationship is not a simple linear one for each child. This is likely due to the multifactorial nature of caries, where the influence of diet is strongly mediated by individual factors like fluoride exposure, oral hygiene effectiveness, and genetic predisposition, which were not captured in the correlation analysis.

A dramatic shift in dental care utilization further complicates this narrative. The post-pandemic group had markedly fewer dental visits, with 63% of children having not seen a dentist in the last year, compared to just 25% in the pre-pandemic group. These results are in agreement with findings from both Brazil [52,53] and the United States [39], where researchers reported significant decreases in pediatric dental procedures and a notable shift away from preventive services. Critically, the motivation for visits shifted from routine check-ups to being primarily driven by pain (61% of visits vs. 17%). This represents a significant regression from proactive prevention to reactive, problem-oriented care. This trend, exacerbated by restricted access during the pandemic [54], meant that opportunities for professional preventive measures like fluoride applications and fissure sealants on newly erupted permanent molars were missed. This lack of professional intervention, combined with the high-sugar diet, created a high-risk environment for the rapid increase in caries observed in the permanent teeth (from 6.8% to 29.6% of teeth affected). The devastating impact of dental pain on a child’s and family’s quality of life, affecting sleep, school, and economic stability, is well-documented and highlights the societal cost of this trend [55,56].

A key finding that may appear controversial is the divergence in caries trends between the primary and permanent dentitions. While caries experience in the permanent dentition increased four-fold, the overall mean dmft score in the primary dentition was paradoxically lower in our post-pandemic cohort. This lower score does not signify better oral health; rather, it reflects a significant shift in disease management. An analysis of the dmft components reveals that the lower total score in the post-pandemic group is driven by a reduction in untreated decayed (d) teeth but a concomitant and significant increase in missing (m) and filled (f) teeth. This finding is consistent with the shift toward pain-driven, problem-oriented care, where treatment for advanced lesions is more likely to be definitive (i.e., restoration or extraction) than monitoring. Therefore, the seemingly “better” dmft score is a clinical marker of a more reactive and less preventive approach to care. In contrast, the alarming rise in the DMFT score is the most direct indicator of the pandemic’s net negative impact. These permanent teeth, particularly the first molars, erupted directly into this high-risk environment during a vulnerable period. A robust body of evidence confirms that newly erupted enamel undergoes a critical, months-long period of post-eruptive maturation, during which it is more porous and significantly more susceptible to demineralization [57]. This physiological vulnerability is compounded by the complex morphology of first molars, whose deep pits and fissures are highly retentive for plaque and are the primary sites for caries initiation in children, making them a key target for preventive measures, such as sealants [58]. The combination of these factors explains the heightened susceptibility of these teeth to the cariogenic challenge identified in our post-pandemic cohort.

The influence of socioeconomic factors, particularly parental education, adds another layer of complexity. As our results statistically confirm, higher parental education was positively correlated with more frequent toothbrushing, a finding consistent with other studies [59,60]. However, this educational advantage did not translate into better dietary control or proactive dental visits, demonstrating that knowledge of one health behavior (hygiene) does not automatically confer protection if other, more powerful risk factors are not controlled. This disconnect highlights the need for interventions that extend beyond simple instructions and address the complex social and environmental factors influencing health behaviors [61]. To more objectively explore the combined influence of these factors, we performed a multiple linear regression analysis. The model confirmed that being in the post-pandemic cohort and having a lower level of maternal education were the most powerful determinants of higher caries experience in the permanent dentition. The finding that diet and brushing were not significant predictors in this specific model does not diminish their clinical importance; instead, it suggests that the ‘post-pandemic cohort’ variable likely functions as a powerful statistical proxy for the entire cluster of negative environmental changes—including the documented dietary shifts, reduced access to care, and other stressors—whose combined effect was the most dominant driver of the adverse oral health outcomes observed.

A crucial consideration is whether the observed increase in caries represents a transient pandemic-related event or the onset of a long-term trend for this pediatric cohort. While some behavioral patterns may normalize, the clinical outcomes indicate a long-lasting negative trajectory. The damage from dental caries in the permanent dentition is cumulative, and an initial lesion at age six begins a lifelong restorative cycle. While modern restorations and crowns exhibit good short-term success, their long-term survival is limited, often leading to a cascade of re-restorations of increasing complexity and cost over an individual’s lifetime [62,63]. A lasting impact on well-being parallels this clinical burden; a high caries experience in childhood is a strong predictor of poorer oral health-related quality of life (OHRQoL) that persists into adulthood [64,65]. This entire negative cascade was initiated by decay developing in highly susceptible, newly erupted molars with immature enamel [66], representing a critical missed opportunity for prevention. Therefore, this cohort is now on a trajectory of poorer long-term oral health and will likely carry an increased lifetime burden of dental disease and its associated costs.

Finally, the data from the COVID-19-specific questionnaire reveal a concerning disconnect between parental perception and clinical reality, a critical barrier to preventive care. A clear majority of post-pandemic parents (60.3%) believed their child’s sugar intake had not changed, a perception directly contradicted by the significant increase in consumption documented in our dietary analysis. This gap suggests a low level of oral health literacy, wherein parents may not recognize the signs of early-stage decay or may underestimate the impact of diet [67,68]. This finding is crucial, as parental awareness is a key driver for seeking preventive care. It reinforces that public health interventions must extend beyond simple instruction; they must actively empower parents to become more accurate assessors of their child’s oral health risks and needs, thereby fostering a more proactive approach to dental care [50].

### 4.1. Limitations

This study has several limitations that should be taken into account when interpreting the findings.

First, the study’s primary limitations stem from its design and sampling methodology. We employed a comparative cross-sectional design rather than a longitudinal one, which prevents the inference of direct causality regarding the pandemic’s impact. This is compounded by the use of different sampling strategies for the two cohorts (stratified WHO Pathfinder vs. convenience). The convenience sampling for the post-pandemic group, in particular, introduces a potential for selection bias, as participating schools may differ systematically from non-participating ones. This inherently limits the generalizability of our post-pandemic findings to the entire rural child population of the region.

Second, limitations related to data collection and reporting must be acknowledged. All behavioral and perceptual data—including diet, oral hygiene, and dental visit history—were collected via parental self-report and are therefore susceptible to both recall and social desirability biases. Furthermore, our protocol for converting ICDAS to DMFT scores included established but non-cavitated dentinal lesions (ICDAS 4) as “decayed.” This sensitive diagnostic threshold, while clinically relevant, may yield higher caries prevalence estimates than studies defining decay solely by the presence of a surface cavity, a critical factor to consider in cross-study comparisons.

Finally, although our statistical analysis included a multiple linear regression model to predict the DMFT score, we did not collect data on all potential unmeasured confounders. For instance, the study did not account for the parents’ own oral health status or long-term dietary habits, which can influence a child’s outcomes through familial and genetic factors. Future research could also employ other models, such as logistic regression, to analyze binary outcomes like the presence or absence of caries.

Despite these limitations, the study has notable strengths. A post-hoc power analysis showed that the study was more than sufficiently powered (>0.99) to detect the main effect observed in the permanent dentition. Its principal strength lies in its unique position as one of the first studies to provide a real-world clinical snapshot comparing pre- and post-pandemic oral health in a vulnerable pediatric population, revealing critical trends with profound implications for public health policy.

### 4.2. Public Health Implications and Future Directions

The findings of this study have significant implications for pediatric health policy in Romania and other middle-income countries. The lessons from other nations are instructive; research from Brazil emphasizes the need to protect children’s access to primary dental care even during crises [53], while experiences in Ghana highlight the importance of community-based programs for reaching rural populations [69]. Building on these lessons and the specific results of international research and our study, we propose to conduct larger scale studies and the following evidence-based recommendations.

First, public health messaging must evolve beyond a singular focus on hygiene. Our study shows that improved brushing was insufficient, and the literature confirms that multi-component programs combining nutritional counseling with toothbrushing promotion are more effective at changing the key behaviors that reduce caries risk [70,71]. Second, the shift to reactive care must be countered by expanding access to preventive services. School-based sealant programs are a proven, high-impact strategy shown to reduce caries risk in permanent molars by up to 94% [72]. Furthermore, the documented success of tele-dentistry and mobile health (mHealth) interventions in reaching rural populations supports the use of outreach models, such as mobile dental clinics, to deliver preventive care where it is needed most [40]. Finally, given that maternal education was a powerful predictor of caries, interventions must be developed to address health literacy and socioeconomic disparities. The evidence shows that programs targeting parental oral health literacy are effective at improving knowledge and preventive behaviors in the home [70,73]. Given the local context, oral health programs targeting parental oral health literacy and education of children for toothbrushing and nutritional counseling are more feasible. Parental oral health literacy is a strong predictor of children oral health behaviors. Educated parents are more likely to implement proper hygiene habits and diet. Improving literacy addresses the root cause. This strategy combined with tooth brushing and nutritional counseling could improve the oral health status and reduce the burden of dental caries.

First, public health messaging must evolve beyond a singular focus on hygiene. Our study shows that improved brushing was insufficient, and the literature confirms that multi-component programs combining nutritional counseling with toothbrushing promotion are more effective at changing the key behaviors that reduce caries risk.

This supports the development of targeted, community-based educational initiatives to empower parents to become more proactive in managing their children’s oral health. Ultimately, building a resilient pediatric oral health system requires a multi-faceted, evidence-based strategy that addresses behavior, access, and education simultaneously.

## 5. Conclusions

This study identifies a significant challenge in post-pandemic pediatric oral health within the specific cohort of rural Romanian children studied. We observed a paradoxical situation in which documented improvements in home oral hygiene practices did not prevent a marked increase in dental caries in the permanent dentition. This adverse clinical outcome coincided with negative shifts in dietary patterns and a sharp decline in access to preventive dental services—factors that appear to have counteracted the benefits of more frequent toothbrushing.

These findings suggest that public health initiatives focused predominantly on a single behavior, such as toothbrushing, may be insufficient to address the multifaceted nature of caries risk in the current environment. An approach that relies heavily on individual hygiene without equally addressing systemic factors shows apparent limitations. Consequently, a strategic evolution of pediatric oral health policies is essential. Future national programs should be broadened to create a more integrated framework, supplementing hygiene education with robust nutritional counseling, implementing measures to encourage proactive dental attendance, and developing targeted initiatives to improve parental health literacy. Adopting such a comprehensive strategy is crucial for effectively mitigating caries risk and securing better long-term oral health outcomes for children in Romania and beyond.

It is important to interpret these findings in light of the study’s main methodological limitations, including the comparative cross-sectional design, the use of a convenience sample for the post-pandemic cohort, and the protocol for converting diagnostic indices. Despite these constraints, this study provides a valuable and detailed snapshot of the complex oral health challenges facing children in the post-pandemic era, underscoring the need for a comprehensive, multi-faceted public health response.

## Figures and Tables

**Table 1:** Protocol for conversion of ICDAS II codes to DMFT scores.

ICDAS Code ^a^	Lesion Description	DMFT Classification ^b^	Rationale
Caries Codes			
0	Sound tooth surface	Not counted	No evidence of caries.
1	First visual change in enamel (only after drying)	Not counted	Non-cavitated, initial-stage lesion.
2	Distinct visual change in enamel	Not counted	Non-cavitated, established lesion.
3	Localized enamel breakdown (no visible dentin)	D = Decayed	Threshold for initial cavitation; aligns with conservative DMFT criteria.
4	Underlying dark shadow from dentin	D = Decayed	Indicates dentin involvement, even if the surface is not fully cavitated.
5	Distinct cavity with visible dentin	D = Decayed	Clear cavitation; matches traditional DMFT classification.
6	Extensive distinct cavity with visible dentin	D = Decayed	Advanced caries; matches traditional DMFT classification.
Outcome Codes			
97	Tooth extracted due to caries	M = Missing	Represents the terminal outcome of the caries process.
Restorative Codes			
3×, 4× ^c^	Tooth restored with a filling (e.g., tooth-colored restoration, amalgam)	F = Filled	Indicates past caries experience that has been treated.

**Note.** Protocol based on the International Caries Detection and Assessment System (ICDAS) II criteria and World Health Organization (WHO) guidelines for epidemiological surveys. ^a^ ICDAS stands for the International Caries Detection and Assessment System. ^b^ DMFT stands for the Decayed, Missing, and Filled Teeth index. ^c^ Represents teeth where the first digit of the two-digit ICDAS code is 3 (tooth-colored restoration) or 4 (amalgam restoration), indicating a surface restored due to previous caries.

**Table 2:** Comparison of mean caries experience (dmft/DMFT) per child between cohorts.

Variable	Group 1 (Pre-Pandemic) n = 77	Group 2 (Post-Pandemic) n = 136	*p*-Value
**Primary Dentition (dmft)**			
Mean dmft score (±SD)	6.11 ± 0.47	4.72 ± 0.31	<0.001
Mean D component (±SD)	5.92 ± 0.47	4.13 ± 0.29	<0.001
Mean M component (±SD)	0.17 ± 0.07	0.35 ± 0.06	<0.001
Mean F component (±SD)	0.01 ± 0.01	0.26 ± 0.05	<0.001
**Permanent Dentition (DMFT)**			
Mean DMFT score (±SD)	0.42 ± 0.08	1.56 ± 0.10	<0.001
Mean D component (±SD)	0.24 ± 0.06	1.40 ± 0.10	<0.001
Mean M component (±SD)	0.16 ± 0.05	0.01 ± 0.01	<0.01
Mean F component (±SD)	0	0.15 ± 0.04	<0.01

**Note.** dmft = decayed, missing, and filled primary teeth; DMFT = Decayed, Missing, and Filled permanent Teeth; D = Decayed; M = Missing; F = Filled; SD = Standard Deviation. Statistical significance was assessed using the independent samples *t*-test.

**Table 3:** Tooth-level analysis of caries distribution and index composition by cohort.

Variable	Group 1 (Pre-Pandemic)	Group 2 (Post-Pandemic)	*p*-Value
**Primary Dentition**	**(Total Teeth = 1085)**	**(Total Teeth = 2401)**	
Total affected teeth, n (%)	470 (43.3%)	643 (26.8%)	<0.001
Index composition, n (%) ^a^			
Decayed (D)	456 (97.0%)	561 (87.2%)	<0.001
Missing (M)	13 (2.7%)	47 (7.3%)	<0.01
Filled (F)	1 (0.2%)	35 (5.4%)	<0.001
**Permanent Dentition**	**(Total Teeth = 456)**	**(Total Teeth = 720)**	
Total affected teeth, n (%)	31 (6.8%)	213 (29.6%)	<0.001
Index composition, n (%) ^a^			
Decayed (D)	19 (61.3%)	191 (89.7%)	<0.001
Missing (M)	12 (38.7%)	2 (0.9%)	<0.001
Filled (F)	0 (0.0%)	20 (9.4%)	0.152

**Note.** Statistical significance was assessed using the chi-square (χ^2^) test for proportions. ^a^ For index composition, the percentage is calculated based on the total number of affected teeth for that dentition and cohort, not the total number of teeth examined.

**Table 4:** Comparison of mean consumption scores for sugary items between cohorts.

**Food/Drink Category**	**Mean Score (2020)**	**Mean Score (2024)**	**t-Statistic**	***p*** **-Value**
Fresh fruit	1.2	2.43	−8.06	<0.001
Cookies, cakes, sweet pies, rolls	1.36	2.34	−7.35	<0.001
Sweets/candies	1.45	2.04	−5.88	<0.001
Sweetened soft drinks	1.94	2.35	−2.45	0.008
Jam/honey	2.21	2.61	−2.44	0.008
Cocoa with sugar/honey	2.84	2.43	2.49	0.007
Tea with sugar/honey (sweetened)	2.44	2.33	0.68	0.249
Milk with sugar/honey	2.58	2.5	0.49	0.313
Chewing gum containing sugar	2.17	1.86	1.62	0.053
Overall mean sweets score (±SD)	1.99 ± 0.079	2.36 ± 0.031	-	<0.001

**Note.** Consumption was rated on a scale from 1 (never) to 6 (several times a day). A higher score indicates more frequent consumption. *t*-tests compare the means between the 2020 and 2024 groups.

**Table 5:** Association between brushing frequency, mean sweets score, and caries experience in primary (dmft) and permanent (DMFT) dentition, by cohort.

Cohort and Brushing Frequency	N	Mean Sweets Score (±SD)	Dentition	Mean dmft ^1^/ DMFT ^2^ (±SD)	Mean Decayed Component (±SD)
Pre-Pandemic					
Once/day	31	2.48 ± 0.95	Primary	6.74 ± 3.34	6.48 ± 3.41
			Permanent	0.35 ± 0.61	0.32 ± 0.54
≥Twice/day	21	2.43 ± 0.81	Primary	4.67 ± 4.56	4.52 ± 4.43
			Permanent	0.33 ± 0.66	0.1 ± 0.3
Post-Pandemic					
Once/day	34	3.59 ± 1.16	Primary	4.44 ± 3.52	3.85 ± 3.2
			Permanent	1.47 ± 1.08	1.32 ± 1.09
≥Twice/day	95	3.37 ± 1.13	Primary	4.93 ± 3.78	4.33 ± 3.57
			Permanent	1.61 ± 1.24	1.47 ± 1.25

**Note.** ^1^ dmft refers to the total primary caries experience (decayed, missing, filled primary teeth). ^2^ DMFT refers to the total permanent caries experience (Decayed, Missing, Filled permanent Teeth). The data presented are descriptive statistics (mean ± standard deviation). The statistical comparisons between brushing frequency groups discussed in the text were conducted using an independent samples *t*-test; full results of these tests are reported in the narrative of the Results section.

**Table 6:** Multiple linear regression predicting DMFT score in permanent dentition.

Variable	B	SE B	β	t	*p*-Value
(Constant)	−0.1	0.43		−0.24	0.812
Maternal education	−0.2	0.06	−0.23	−3.54	<0.001
Sweets consumption score	−0.07	0.07	−0.07	−0.99	0.324
Study cohort (post-pandemic)	1.34	0.2	0.5	6.78	<0.001
Brushing frequency (≥Twice/day)	0.1	0.17	0.04	0.58	0.566

**Note.** B = Unstandardized coefficient; SE B = standard error of the coefficient; β = standardized coefficient. The reference category for study cohort is ‘pre-pandemic’, and that for brushing frequency is ‘once/day’.

**Table 7:** Parental perceptions of the COVID-19 pandemic’s impact (post-pandemic cohort).

Domain & Response Categories	n	(%)
**1. Difficulties Accessing Dental Services (n = 136)**		
Yes, access was difficult ^a^	47	(34.6)
No, child did not require services	78	(57.3)
No, we always had access	11	(8.1)
**2. Financial Challenges Impacting Dental Care (n = 109) ^b^**		
Yes, finances were a challenge ^c^	29	(26.6)
No, finances did not impact care	80	(73.4)
**3. Change in Child’s Sweets Consumption (n = 136)**		
Increased ^d^	36	(26.5)
Decreased ^e^	18	(13.2)
Stayed the same	82	(60.3)
**4. Development of Stress-Related Oral Habits (n = 133)**		
Yes, habits developed ^f^	63	(47.4)
No, child did not develop habits	70	(52.6)
**5. Perceived Cause of Recent Dental Problems (n = 109)**		
Caused by pandemic-related delays ^g^	95	(87.2)
Not related to the pandemic	14	(12.8)

**Note.** Percentages (%) are calculated based on the number of valid responses (n) for each domain. ^a^ Category combines original responses for “Yes, during and after the pandemic” and “Yes, during the pandemic (2020–2022)”. ^b^ Denominator (n) is less than the total post-pandemic cohort size (*n* = 136) due to missing responses for this question. ^c^ Category combines original responses for “Yes, during and after the pandemic” and “Yes, only during the pandemic (2020–2022)”. ^d^ Category combines original responses for “Slightly increased” and “Significantly increased”. ^e^ Category combines original responses for “Slightly decreased” and “Significantly decreased”. ^f^ Category combines original responses for “Yes, stress-related oral habits developed during the pandemic and are still present” and “Yes, stress-related oral habits developed during the pandemic but then ceased”. ^g^ Category combines original responses for “Likely caused by pandemic-related delays” and “Surely caused by pandemic-related delays”.

## Data Availability

The datasets generated and analyzed during the study are available from the corresponding author upon reasonable request.

## Abbreviations

The following abbreviations are used in this manuscript:

COVID-19	Coronavirus Disease 2019
CPI	Community Periodontal Index
D	Decayed
dmft	decayed, missing, and filled primary teeth
DMFT	Decayed, Missing, and Filled Teeth
ECC	Early Childhood Caries
F	Filled
ICDAS	International Caries Detection and Assessment System
M	Missing
mHealth	Mobile Health
OHRQoL	Oral Health-Related Quality of Life
SARS-CoV-2	Severe Acute Respiratory Syndrome Coronavirus 2
SD	Standard Deviation
SES	Socioeconomic Status
U.S.	United States
WHO	World Health Organization
χ^2^	chi-square
